# Gdx mediates low-affinity Cs⁺/H⁺ antiport and confers cesium resistance in *Escherichia coli*

**DOI:** 10.1016/j.engmic.2025.100251

**Published:** 2025-12-18

**Authors:** Daiki Kojima, Masahiro Ito

**Affiliations:** a Graduate School of Life Sciences, Toyo University, Asaka, Saitama 351-8510,Japan; b Bio-resilience research project (BRRP), Toyo University, Asaka, Saitama 351-8510, Japan; c Bio-Nano Electronics Research Center, Toyo University, Kawagoe, Saitama 350-8585, Japan

**Keywords:** Cesium-resistant mechanism, *Escherichia coli*, SugE, Small multidrug resistance (SMR)

## Abstract

Following the Fukushima Daiichi nuclear power plant accident, radioactive cesium was released into the environment, prompting intensified efforts to identify cesium-resistant microorganisms. During these studies, we isolated a cesium-resistant *Escherichia coli* strain, designated ZX-1, which exhibits remarkable tolerance to cesium concentrations exceeding 700 mM. As no prior reports of cesium-resistant *E. coli* exist, this finding suggests the presence of a previously unrecognized resistance mechanism. This study aims to elucidate the molecular basis of cesium resistance in ZX-1.

RNA-seq analysis comparing ZX-1 with its parental strain, the commercial *E. coli* Mach1™, revealed constitutive upregulation of the guanidinium exporter gene *gdx* in ZX-1. Reanalysis of the whole-genome sequence identified a 20-bp deletion upstream of the *gdx* open reading frame, likely disrupting formation of the guanidinium riboswitch P2 loop and resulting in constitutive *gdx* expression.

To evaluate *gdx* function, the gene was cloned into the expression vector pBAD24 and expressed in *E. coli*. The resulting *gdx*-expressing strain exhibited even greater cesium resistance than ZX-1. Functional assays demonstrated that this strain mediates not only guanidinium/H⁺ antiport activity but also Cs⁺/H⁺ antiport activity. Cesium resistance was further enhanced in the presence of guanidinium, consistent with riboswitch-mediated induction of *gdx*.

Collectively, these findings provide evidence for a novel cesium efflux mechanism in *E. coli* and uncover an unexpected role of the guanidinium exporter Gdx in cesium export. These insights may facilitate the discovery of additional cesium-resistant microorganisms and broaden the potential for future applications.

## Highlights

• gdx is strongly upregulated in the Cs⁺-resistant strain ZX-1.
• A 20-bp deletion in the riboswitch P2 loop causes constitutive gdx expression.
• Gdx exports Cs⁺ via H⁺ antiport, at ∼500-fold lower affinity than for Gdm⁺.
• Gdm⁺ boosts growth via riboswitch-mediated induction of gdx under Cs⁺ stress.
• Gdx expression lowers intracellular Cs⁺ and preserves K⁺ homeostasis.

## Graphical abstract

**Figure:**
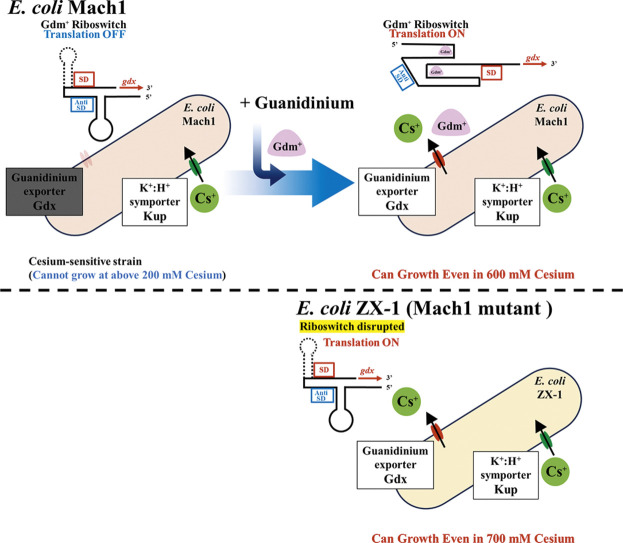

## 1 Introduction

The Fukushima Daiichi nuclear power plant accident in 2011 resulted in the release of a substantial amount of radionuclides, including radioactive cesium isotopes (Cs-134 and Cs-137), into the environment [1,2]. These radioactive Cs isotopes accumulated in the surface layers of nearby soils, prompting large-scale soil removal operations. However, such physical decontamination methods pose various challenges, including high financial costs and the need for public acceptance regarding the storage of contaminated soils [3,4].

Since the accident, research on the bioremediation of Cs-contaminated environments has intensified. Several studies have investigated Cs^+^ adsorption and extraction using various plant species [[5], [6], [7]]. In addition, several Cs^+^-resistant microorganisms have been identified. For instance, bacterial strains CS98 and CS402 have been reported to accumulate Cs^+^ at maximum levels of 52.0 μmol/g and 18.8 μmol/g dry weight, respectively [8]. *Arthrobacter* sp. KMSZP6 was shown to accumulate 9612 mg/g dry weight of Cs^+^ after 12 h of cultivation in medium containing 75 mM CsCl [9]. Similarly, *Arthrobacter globiformis* 151B was reported to mediate both absorption and accumulation of Cs^+^ and hexavalent chromium [10].

A Cs^+^-resistant strain, *Microbacterium* sp. TS-1, capable of tolerating up to 1200 mM CsCl, was isolated from a homogenized sample of jumping spider [11]. This strain was shown to extrude intracellular Cs^+^ via a Cs⁺/H⁺ antiporter, CshA, belonging to the major facilitator superfamily (MFS) [12]. Further studies revealed that the active intracellular uptake of magnesium ions is also critical for survival under low-Cs^+^ stress conditions [13], and that magnesium supplementation enhances Cs^+^ resistance in various microbial species [14]. This phenomenon is hypothesized to result from magnesium binding to ribosomes, thereby stabilizing them against excess Cs^+^-induced stress.

Cs is physiochemically similar to potassium (K) and is known to follow K^+^-associated transport pathways in living organisms [15,16]. In *Escherichia coli*, Cs^+^ influx has been shown to occur via the major K^+^ transporter Kup, leading to intracellular Cs^+^ accumulation and K^+^ efflux for turgor maintenance [17]. This K^+^ depletion ultimately impairs cell growth under Cs^+^ stress.

A recent study reported an *E. coli* strain, ZX-1, exhibiting Cs^+^ resistance at concentrations exceeding 700 mM—an unprecedented finding [18]. Whole-genome sequencing revealed that ZX-1 carries nonsynonymous mutations in three genes: the flagellar basal-body rod protein gene *flgG*, the ribosomal bS6 modification enzyme gene *rimK*, and a gene encoding a phage lysis regulatory protein LysB. Moreover, intracellular Cs^+^ measurements demonstrated that ZX-1 maintains lower internal Cs^+^ levels compared to its parental strain Mach1™ under Cs^+^ stress. However, the precise mechanism underlying the Cs^+^ resistance of ZX-1 remained unresolved.

The guanidinium exporter Gdx (formerly known as SugE) is a member of the small multidrug resistance (SMR) family and is characterized as a four-transmembrane efflux protein. It has been actively studied in the context of hospital-acquired bacterial infections [[19], [20], [21], [22], [23], [24]]. Although Gdx belongs to the SMR family, no reports have demonstrated that it exports conventional antimicrobial drugs [20,[23], [24], [25]]. Instead, Gdx exhibits high substrate specificity and is known to mediate the efflux of guanidinium—a bactericidal compound—as well as certain quaternary ammonium compounds [19,21,24]. To date, however, Gdx has never been linked to metal-cation efflux, and no study has suggested a relationship between Gdx and Cs⁺ physiology. This raised the possibility that the Cs⁺-resistant strain ZX-1 might utilize an unexpected mechanism involving Gdx.

The present study aims to elucidate the Cs^+^ resistance mechanism of ZX-1 by RNA-seq-based transcriptomic analysis under Cs⁺ stress, thereby uncovering a previously unreported Cs⁺-resistance pathway in *E. coli*.

## 2 Materials and methods

### 2.1 Bacterial strains and plasmids

The bacterial strains and plasmids used in this study are listed in Table 1. The plasmid pBAD_Gdx was constructed by amplifying the *gdx* gene from the genomic DNA of *E. coli* strain W3110 using the primers gdx-EcoRI-F (5′–CCCGAATTCATGTCCTGGATTATCTTAGTTATTGC–3′) and gdx-XbaI-R (5′–CCCCCCTCTAGATTAGTGAGTGCTGAGTTTCAGAC–3′). PCR amplification was performed using PrimeSTAR GXL DNA Polymerase (Takara Bio, Japan) according to the manufacturer’s instructions. The PCR product was digested with EcoRI and XbaI and ligated into EcoRI/XbaI-digested pBAD24 vector using T4 DNA ligase. The organization of the pBAD_Gdx construct, including the *gdx* insert under control of the P_BAD_ promoter, is shown in Supplementary Figure S1.

**Table 1:** Bacterial strains and plasmids are used in this study.

Strains/Plasmids		Genotype	References
Strains	Mach1™	F^-^, [φ80*lac*ZΔM15], Δ*lac*X74, *hsd*R, (r_K_^–^, m_K_^+)^ , Δ*rec*A1398, *end*A1, *ton*A	Thermo Fisher
	ZX-1	Ap^R^, Cs-resistant strains	[18]
	KNabc	TG1 (Δ*nhaA* Δ*nhaB* Δ*chaA*)	[26]
Plasmids	pBAD24	Cloning expression vector, P_BAD_ promoter, Ap^R^	[27]
	pBAD_CshA	pBAD24 + TS_*cshA*, Ap^R^	[12]
	pBR322ΔAp	Cloning vector, Tet^R^	[18]
	pBAD_Gdx	pBAD24 + *gdx,* Ap^R^	This study

### 2.2 Growth media and conditions

*E. coli* strains Mach1™ and ZX-1 were cultured in Luria–Bertani (LB) medium composed of 10 *g*/L tryptone, 5 *g*/L yeast extract, and 10 *g*/L NaCl, supplemented with various concentrations of cesium chloride (CsCl), and incubated at 37 °C with shaking at 200 rpm. The strain KNabc was cultured in LBK medium, in which NaCl was replaced with 6 *g*/L KCl, and the pH was adjusted to 7.5 by 6 N KOH. Cultures were incubated under the same conditions with varying concentrations of CsCl. When antibiotic selection was required, tetracycline (25 μg/mL) or ampicillin (100 μg/mL) was added to the medium. For arabinose-inducible gene expression, l-arabinose was added at a final concentration of 2 % (w/v). Bacterial growth was monitored by measuring optical density at 600 nm (OD₆₀₀) using a UV–visible spectrophotometer (Shimadzu, Japan).

### 2.3 Transcriptome analysis of *E. coli* strains mach1™ and ZX-1

Whole-genome analysis of the E. coli strain ZX-1 has already been completed, and SNPs relative to the parental strain Mach1 were reported [18]. In the previous analysis, 11 SNPs and 2 deletions were detected. The sequence data generated are available in the DNA Data Bank of Japan (DDBJ) Sequence Read Archive under accession numbers DRA017249 (ZX-1) and DRA017250 (Mach1™). To comprehensively determine how the ZX-1 mutations affect the expression of individual genes, an RNA-seq–based screen for Cs⁺-resistance genes was conducted. For transcriptome sampling, Mach1™/pBAD24 was cultured under CsCl-free conditions until OD₆₀₀ reached 0.4. ZX-1/pBR322ΔAp was cultured under both CsCl-free and 700 mM CsCl-supplemented conditions until OD₆₀₀ reached 0.4.

#### 2.3.1 RNA extraction and library preparation

Cells were mechanically disrupted using Buffer RLT with β-mercaptoethanol and a Multi-Beads Shocker (Yasui Kikai, Japan). Total RNA was extracted using the RNeasy Mini Kit on a QIAcube Connect (Qiagen) following the manufacturer’s instructions. RNA quantity was measured using the QuantiFluor RNA System (Promega) on a Quantus Fluorometer, and RNA integrity was assessed with the Agilent High Sensitivity RNA Kit on a 5200 Fragment Analyzer (Agilent Technologies). Ribosomal RNA was depleted using riboPOOL (siTOOLs Biotech), and strand-specific RNA-seq libraries were constructed using the MGIEasy RNA Directional Library Prep Set (MGI Tech). Library quantity and quality were confirmed using a Qubit 3.0 Fluorometer (Thermo Fisher Scientific), Agilent 2100 Bioanalyzer, and Fragment Analyzer (Agilent Technologies). DNA libraries were circularized and converted into DNA nanoballs (DNBs) using MGIEasy kits (MGI Tech). Paired-end sequencing (2 × 150 bp) was performed on a DNBSEQ-G400 platform (MGI Tech).

#### 2.3.2 Data processing and transcriptome analysis

Adapters were removed using Cutadapt (v4.0), and low-quality reads (*Q* < 20) or reads <75 bp were filtered with Sickle (v1.33). Clean reads were mapped to the *E. coli* reference genome (GCF_000005845.2) with Bowtie2 (v2.5.0), followed by sorting and indexing with Samtools (v1.17). Gene-level counts were obtained with featureCounts (v2.0.3). For visualization and within-sample composition, expression was summarized as transcripts per million (TPM).

This RNA-seq analysis was exploratory with *n* = 1 per condition; therefore, no statistical testing of differential expression was performed. edgeR (v3.36.0) after DEGES normalization in TCC (v1.34.0) was used only to compute normalization factors, log-scale summaries, and effect sizes (log₂ fold changes) for candidate prioritization; no p-values or FDR are reported. Candidates referenced in the Results were filtered by prespecified heuristics (e.g., TPM ≥ 10 in either condition and |log₂FC| ≥ 2).

Functional annotation used KEGG (BLASTN, v2.15.0) and Gene Ontology (DIAMOND, v2.1.6). Because of *n* = 1, no over-representation/enrichment tests were conducted. RNA-seq processing details are provided in Supplementary Table 1.

### 2.4 Manual extraction and comparison of gdx gene and upstream sequences

The genomic sequences of the *gdx* gene (position: 1155,586–1155,903, + strand) and its 200 bp upstream region were manually extracted from the assembled FASTA files of strains ZX-1 and Mach1™. These genome assemblies had been reported previously [18]. The sequence extraction was performed using a custom Python script based on Biopython (v1.85). The extracted sequences were then manually compared to identify sequence differences, including upstream deletions. Sequence data are available in Supplementary Table 2.

### 2.5 Structural prediction of gdx using alphafold3 and deeptmhmm

The amino acid sequence of Gdx from *E. coli* strain K-12 (W3110) (NCBI accession ID: P69937) was used for structural prediction. The three-dimensional structure of Gdx was generated using the AlphaFold3 web server (https://deepmind.google/science/alphafold/) [28]. and the membrane topology was predicted using DeepTMHMM, web server (v1.0.44, https://dtu.biolib.com/DeepTMHMM/) [29]. The predicted structure and membrane topology are shown in Supplementary Figure S2.

### 2.6 Expression of gdx and cesium tolerance assay in E. coli

The plasmid pBAD_Gdx was introduced into *E. coli* Mach1™ to generate the strain Mach1™/pBAD_Gdx. For preculture, Mach1™/pBAD_Gdx was grown in LB medium at 37 °C for 16 h. A 10 μL aliquot of the preculture was then inoculated into 2 mL of fresh LB medium with or without 2 % (w/v) l-arabinose, followed by incubation at 37 °C for 8 h to induce gene expression.

Subsequently, 10 μL of each culture was inoculated into 2 mL of LB medium containing either 0 mM or 700 mM CsCl, with or without l-arabinose, and cultured at 37 °C for 16 h. Cell growth was evaluated by measuring the optical density at OD₆₀₀.

Additionally, the strains Mach1™/pBAD_Gdx, Mach1™/pBAD24 (empty vector control), and ZX-1/pBR322ΔAp were each precultured under the same conditions in LB medium containing 2 % (w/v) l-arabinose. After preculture, 10 μL of each culture was inoculated into 2 mL of LB medium containing l-arabinose (2 % w/v) and a range of CsCl concentrations (0 to 700 mM). Following 16 h of incubation at 37 °C, OD₆₀₀ was measured to assess Cs^+^ tolerance.

### 2.7 Preparation of everted membrane vesicles and measurement of cation/h⁺ antiport activity in gdx-overexpressing E. coli

The plasmid pBAD_Gdx was introduced into *E. coli* strain KNabc, which lacks the major Na⁺/H⁺ antiporters NhaA, NhaB, and ChaA. For preculture, the transformant was grown in 2 mL of LBK medium at 37 °C with shaking at 200 rpm for 8 h. Subsequently, 1 mL of the culture was inoculated into a 500 mL Erlenmeyer flask containing 200 mL of LBK medium supplemented with 2 % (w/v) l-arabinose and ampicillin (100 µg/mL), and incubated at 37 °C for 16 h with shaking at 200 rpm.

Cells were harvested by centrifugation at 10,400 × *g* for 10 min and used for the preparation of everted membrane vesicles, following the method described in [26]. Briefly, the cell pellet was washed with 25 mL of TCDG buffer (10 mM Tris–HCl, pH 8.0, 5 mM MgCl₂, 10 % glycerol, 140 mM choline chloride, and 1 mM dithiothreitol). The washed cells were resuspended in 25 mL of the same buffer supplemented with a protease inhibitor cocktail (half tablet; Roche), 25 µL of 100 mM phenylmethylsulfonyl fluoride (PMSF), and 4 mg of DNase I. Cell disruption was performed using a high-pressure homogenizer (Glen Mills, USA) at 10,000 psi. Unbroken cells were removed by centrifugation at 10,400 × *g* for 10 min at 4 °C, and the supernatant was subjected to ultracentrifugation at 208,400 × *g* for 1 h at 4 °C. The resulting membrane pellet was resuspended and homogenized in 1 mL of TCDG buffer.

The protein concentration of the prepared vesicles was determined by the Lowry method with bovine serum albumin (BSA) as the standard [30].

Cation/H⁺ antiport activity was measured as in [31]. using a fluorescence spectrophotometer (Hitachi High-Tech, Japan). The reaction mixture contained 2 mL of antiport activity assay buffer (140 mM choline chloride, 50 mM Bis-Tris propane; pH adjusted to 8.0, 8.5, or 9.0 with H₂SO₄), 0.7 µL of 1 M acridine orange, and everted membranes corresponding to 66 µg of protein. After the baseline fluorescence stabilized, 10 µL of 0.5 M succinate (pH 8.0 with Tris base) was added as a respiratory substrate. Upon observing fluorescence quenching, cation substrates—guanidinium hydrochloride (GdmCl), NaCl, KCl, CsCl, or CaCl₂—were added at the indicated concentrations. Finally, 5 µL of 4 M (NH₄)₂SO₄ was added to terminate the measurement.

Antiport activity (percent dequenching) was calculated as the ratio of the fluorescence increase upon cation addition to the total increase observed between succinate addition and (NH₄)₂SO₄ addition. Apparent *K*ₘ values for each substrate were determined from Lineweaver–Burk plots generated using substrate concentration versus antiport activity data. For GdmCl and CsCl, antiport activity was assayed at pH 8.0, 8.5, and 9.0. Activity was considered detected when the initial dequenching rate exceeded the limit of detection (LOD), defined as the mean blank rate + 3 × SD (*n* = 3).

### 2.8 Measurement of intracellular cs⁺ and K⁺ concentrations in gdx-expressing E. coli under cs⁺ stress

Strains ZX-1/pBR322ΔAp, Mach1™/pBAD24, and Mach1™/pBAD_Gdx were used, and samples were prepared as described by Ito et al. [32]. Each strain was precultured in 2 mL of LB medium containing 2 % (w/v) l-arabinose together with the appropriate antibiotic (25 µg/mL tetracycline for ZX-1/pBR322ΔAp; 100 µg/mL ampicillin for Mach1™/pBAD24 and Mach1™/pBAD_Gdx). Cultures were then transferred to 500 mL Erlenmeyer flasks containing 100 mL of LB medium with the same antibiotic and 2 % (w/v) l-arabinose and incubated until the turbidity reached an OD₆₀₀ of ∼0.4. Cells were subsequently exposed for 1 h to one of three conditions: CsCl free, 200 mM CsCl, or 700 mM CsCl.

Cells were collected by centrifugation at 3000 × *g* for 10 min at 25 °C. Pellets from the CsCl free and 200 mM CsCl conditions were washed twice with 300 mM sucrose solution. Because pelleting was inefficient after exposure to 700 mM CsCl, cells from this condition were collected and washed on a PTFE membrane filter (0.2 µm pore size; Merck). An aliquot (100 µL) from the first wash fraction was taken for protein quantification by the Lowry method [30].

Washed cell pellets were disrupted by adding 5 mL of 5 % (w/v) trichloroacetic acid (TCA) and heating at 100 °C for 10 min. After centrifugation at 10,400 × *g* for 10 min at 4 °C, the supernatants were collected, and Cs⁺ and K⁺ concentrations were determined with a flame photometer (BWB; Ogawa Seiki, Japan). Intracellular ion concentrations were normalized to cell volume by estimating volume from protein content, assuming that 1 mg of cellular protein corresponds to 3 µL of cell volume.

### 2.9 Cesium tolerance assay of E. coli mach1™ in LB medium containing guanidinium

Mach1™/pBAD24 was precultured in 2 mL of LB medium at 37 °C with shaking at 200 rpm for 8 h. After preculture, 1 mL of the culture was inoculated into fresh LB media containing various concentrations of CsCl, with or without 10 mM GdmCl. The cultures were incubated at 37 °C with shaking at 200 rpm for 16 h. Cell growth was assessed by measuring the optical density at OD₆₀₀.

### 2.10 Alignment of E. coli-derived gdx and homologous proteins from other bacterial species

Homologous sequences of the small multidrug resistance (SMR) family of drug/metabolite exporters were collected across Bacteria and Archaea, focusing on the Gdx protein (previously annotated as SugE). After preliminary filtering, *E. coli* K-12 Gdx (UniProt: P69937) was retained as a required reference, and species-level redundancy was removed by keeping a single representative per species. The final primary dataset comprised 50 taxa (15 archaeal and 35 bacterial).

Multiple sequence alignment was performed on the Galaxy platform [33]. using MAFFT [34,35]. with protein sequences as input and the l-INS-i strategy (equivalent to –localpair –maxiterate 1000; MAFFT version 7.5.26 as provided by the Galaxy tool wrapper). Low-information and gap-rich columns were trimmed with trimAl in automated1 mode (trimAl version 15.0) [36]. The trimmed alignment was then submitted to IQ-TREE (web server; https://iqtree.github.io/) for maximum-likelihood inference [37]. ModelFinder with automatic model selection (MFP) was enabled, and branch support was assessed with 2000 ultrafast bootstrap (UFBoot) replicates and 2000 SH-aLRT replicates. The resulting tree was visualized with iTOL [38].

## 3 Results

### 3.1 Exploratory transcriptomic features of ZX-1 (effect-size–based candidate prioritization)

We compared the transcriptomes of ZX-1 and its parental strain Mach1™ as an exploratory RNA-seq (one biological replicate per strain; OD₆₀₀ = 0.4) to prioritize candidates for follow-up experiments. No statistical testing was performed; differential expression was summarized by effect sizes (log₂ fold change; log₂FC) and TPM and used solely for hypothesis generation.

Because the comparison involved different plasmid backgrounds (Mach1™/pBAD24 vs. ZX-1/pBR322ΔAp), we refrain from mechanistic interpretation for pathways likely sensitive to vector context (e.g., arabinose metabolism).

Within this framework, top candidates included *gdx* (guanidinium exporter; log₂FC = 6.08), *gltS* (Na⁺/glutamate symporter; log₂FC = 6.57), and *cysI*/*D*/*J* (cysteine metabolism; log₂FC = 4.11–4.43). These genes met TPM ≥ 10 in either strain and |log₂FC| ≥ 2 and were ranked by effect size.

An additional exploratory comparison of ZX-1 grown without or with 700 mM CsCl (*n* = 1 each) revealed large effect-size changes for *kdpFABC* (high-affinity K⁺ uptake) and *rmf* (ribosome modulation factor), consistent with K⁺ homeostasis and translational control under Cs⁺ stress. However, statistical significance was not assessed due to *n* = 1. Subsequent functional analyses were focused on *gdx*.

### 3.2 High expression of gdx in ZX-1 is caused by a deletion in the upstream regulatory region

RNA-seq analysis identified *gdx* as specifically upregulated in strain ZX-1, and experiments were therefore directed toward this gene. This focus was motivated by prior observations suggesting that the ZX-1 phenotype involves a mechanism that maintains low intracellular Cs⁺ concentrations [18]. and by the established role of Gdx in transporting monovalent cations [39].

**Fig. 1:**
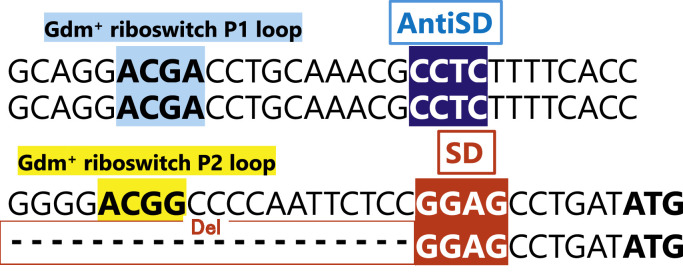
Upstream sequence of the gdx gene in *E. coli* strains Mach1 and ZX-1. A 64 bp region upstream of the *gdx* start codon (ATG) is shown for both strains. In ZX-1, a 20 bp deletion is present from 30 bp to 11 bp upstream of the *gdx* open reading frame (ORF). The ACGA and ACGG motifs (blue and yellow) represent the P1 and P2 loops of the guanidinium riboswitch, respectively. The GGAG motif (red) indicates the Shine-Dalgarno (SD) sequence. The sequences and positions of the P1 and P2 loops are based on reference [21].

As described in the Methods section, the complete genome sequence of strain ZX-1 has already been determined and is publicly available [18]. In this study, reanalysis focused on the *gdx* gene (position: 1155,586–1155,903, + strand) and the 200 bp upstream flanking regions. The analysis revealed a 20 bp deletion (delAGAATTGGGGCCGTCCCCGG, + strand) located 30 bp to 11 bp upstream of the *gdx* open reading frame (ORF) (Fig. 1). This deletion is presumed to affect the upstream regulatory element, leading to the constitutive high-level expression of *gdx* observed in ZX-1.

### 3.3 Overexpression of gdx enhances cesium tolerance in E. coli

To illustrate the structural background of Gdx, we predicted the 3D structure and transmembrane topology of *E. coli* K-12 Gdx using AlphaFold3 and DeepTMHMM. Both predictions consistently showed that Gdx adopts the characteristic SMR-like four-transmembrane-helix architecture, with four predicted transmembrane segments (Figure S2).

**Fig. 2:**
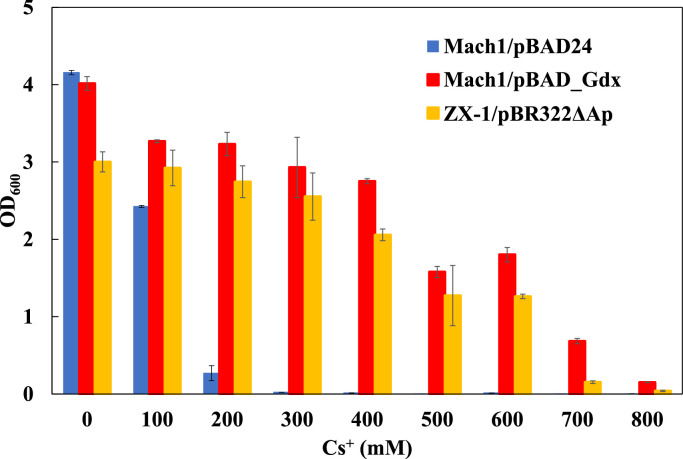
Cesium resistance assay of *E. coli* strains including Guanidinium exporter Gdx-expressing strain. Bar graphs represent OD₆₀₀ turbidity of *E. coli* Mach1™/pBAD24 (blue), Mach1™/pBAD_Gdx (red), and ZX-1/pBR322ΔAp (yellow) cultured under various cesium concentrations (mM), indicated on the x-axis. The y-axis shows OD₆₀₀ after 16 h of cultivation. Error bars indicate the standard deviation of three independent experiments.

Each *E. coli* strain was cultured in LB medium supplemented with CsCl at concentrations ranging from 0 to 800 mM at 37 °C for 16 h. When the strain Mach1™ was transformed with the pBAD_Gdx plasmid, Cs^+^ tolerance was observed only upon induction with 2 % (w/v) l-arabinose (Figure S3). The Cs^+^-sensitive control strain, Mach1™/pBAD24, exhibited growth inhibition at 300 mM CsCl. The Cs^+^-resistant strain ZX-1 showed severely impaired growth at 800 mM CsCl, with an OD₆₀₀ of 0.04. In contrast, the Gdx-overexpressing strain demonstrated enhanced Cs^+^ tolerance, surpassing that of ZX-1, and reached an OD₆₀₀ of 0.15 under the same 800 mM CsCl condition (Fig. 2).

### 3.4 Gdx functions as an antiporter for both guanidinium and cesium ions

Cation/H⁺ antiport activity of Gdx was assessed using everted membrane vesicles and a fluorescence dequenching assay. Under pH 9.0, no Na⁺-coupled antiport activity was detected at 30 mM (Figure S4). Antiport activity toward K⁺ and Ca²⁺ was observed in vesicles from both KNabc/pBAD_Gdx and the vector control KNabc/pBAD24; however, no statistically significant differences between strains were detected at the tested concentrations (*P* ≥ 0.05) (Figures S4 and S5).

**Fig. 3:**
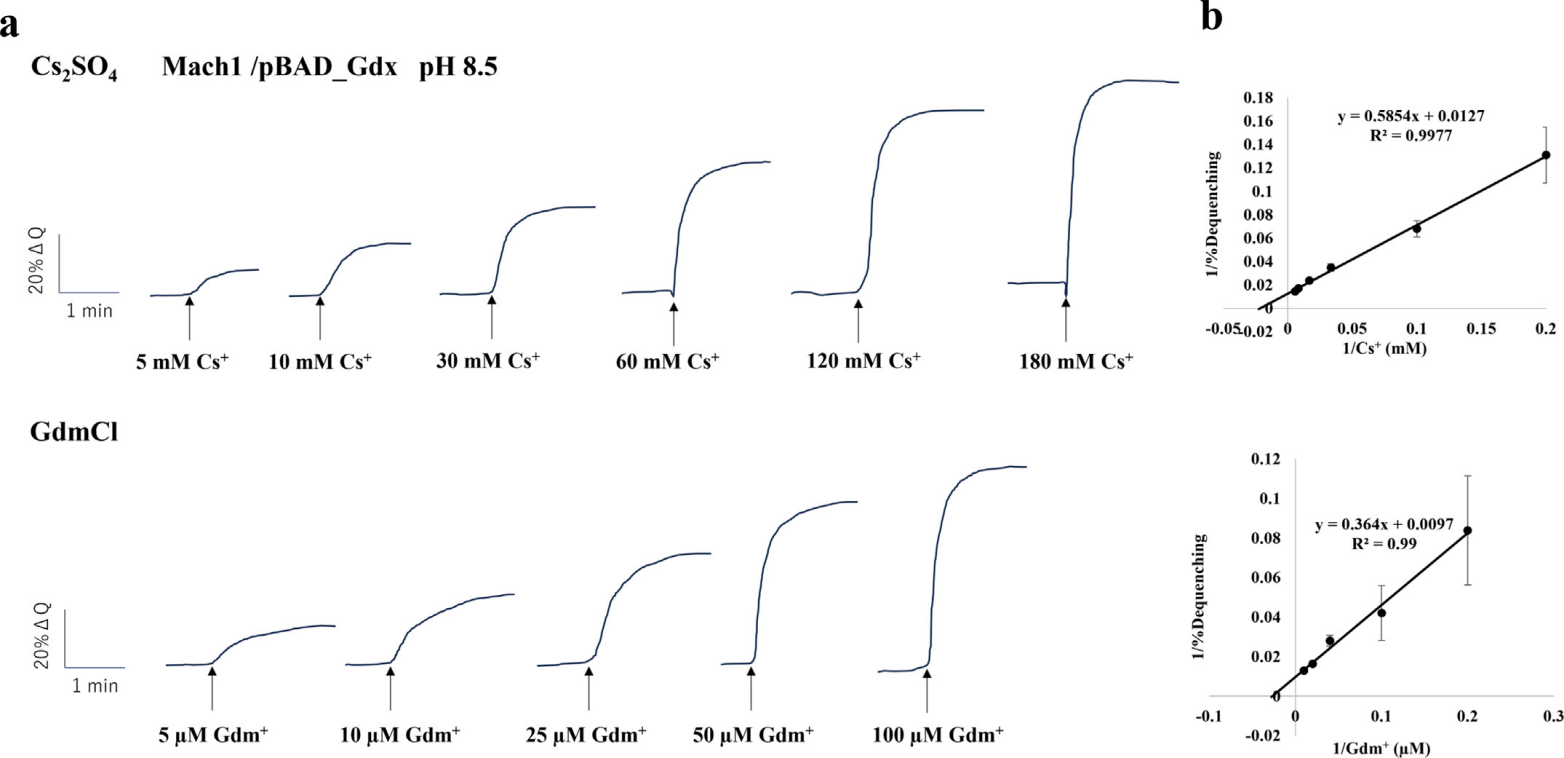
Measurement of Cs⁺/H⁺ and Gdm⁺/H⁺ antiport activities of everted membrane vesicles from *E. coli* KNabc/pBAD_Gdx and corresponding Lineweaver–Burk plots. (a) Representative fluorescence quenching traces from three independent experiments are shown. The upper panel shows Cs⁺/H⁺ antiport activity and the lower panel shows Gdm⁺/H⁺ antiport activity. Arrows indicate the timing of Cs⁺ or Gdm⁺ addition at varying concentrations. (b) Lineweaver–Burk plots were generated from the reciprocal values of antiport activity and substrate concentration (Cs⁺ or Gdm⁺) at pH 8.5. The y-axis represents the reciprocal of the antiport activity, and the x-axis represents the reciprocal of the cation concentration. Error bars represent standard deviations from three independent experiments.

By contrast, under pH 8.0, 8.5, and 9.0, robust antiport activity toward guanidinium (Gdm⁺) and cesium (Cs⁺) was detected only in Gdx-expressing vesicles (Figs. 3, S6, and S7). Lineweaver–Burk plots based on the activity–substrate relationships were used to estimate apparent *K*ₘ values. LB plots are shown at pH 8.5 in Fig. 3 and at pH 8.0/9.0 in Supplementary Figures S6 and S7. For Gdm⁺, the apparent *K*ₘ values were 77.0 µM at pH 8.0, 37.5 µM at pH 8.5, and 29.2 µM at pH 9.0 (Figs. 3, S6, and S7). For Cs⁺, the apparent *K*ₘ values were 65.8 mM at pH 8.0, 46.1 mM at pH 8.5, and 15.1 mM at pH 9.0 (Figs. 3, S6, and S7). These results indicate H⁺-coupled antiport by Gdx for both Gdm⁺ and Cs⁺, with higher apparent affinity at more alkaline pH. Table 2 summarizes antiport activities of the transformants used in this study.

**Table 2:** Cation/H⁺ Antiport Activity of KNabc/pBAD_Gdx.

**Substrate**	**Activity**	***K*_m_ value (pH 8.5)**
Gdm^+^	Detected	37.5 µM
Cs^+^	Detected	46.1 mM
K^+^	aND	bNE
Na^+^	ND	NE
Ca^2+^	ND	NE

a ND: no activity detected above background (signal < LOD).

b NE: parameter could not be estimated because the antiport signal was below the reliable fitting threshold and did not show Michaelis–Menten saturation within the tested range.

### 3.5 Gdx-Expressing cells maintain lower intracellular cesium under cesium conditions

**Fig. 4:**
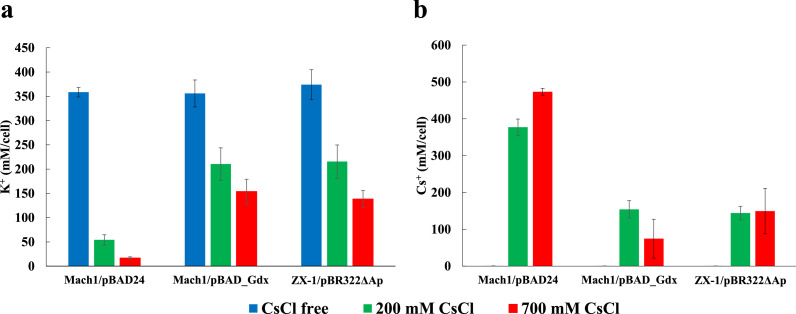
Intracellular K⁺ (a) and Cs⁺ (b) concentrations of *E. coli* strains cultured under each cesium conditions. Bar colors indicate cesium conditions during cultivation: CsCl-free (blue), 200 mM CsCl (green), and 700 mM CsCl (red). The y-axis shows intracellular K⁺ or Cs⁺ concentration normalized to estimated cell volume (assuming 1 mg cellular protein corresponds to 3 µL)[31]. The x-axis lists strain names. Error bars represent standard deviations from three independent experiments.

In cultures without added CsCl, intracellular K⁺ concentrations were approximately 358.5 mM in Mach1™/pBAD24, 356.0 mM in Mach1™/pBAD_Gdx, and 374.0 mM in ZX-1/pBR322ΔAp. Upon exposure to 200 mM CsCl, intracellular K⁺ concentrations were ∼54.2 mM, 210.6 mM, and 215.6 mM, respectively. Under 700 mM CsCl, the values were ∼16.9 mM, 153.8 mM, and 138.4 mM, respectively (Fig. 4a).

Intracellular Cs⁺ concentrations under 200 mM CsCl were ∼377.1 mM in Mach1™/pBAD24, 154.2 mM in Mach1™/pBAD_Gdx, and 144.0 mM in ZX-1/pBR322ΔAp; under 700 mM CsCl they were ∼473.5 mM, 74.6 mM, and 149.3 mM, respectively (Fig. 4b). These data indicate that both Mach1™/pBAD_Gdx and ZX-1/pBR322ΔAp maintain lower intracellular Cs⁺ levels and attenuate the decline in intracellular K⁺ under Cs^+^ stress, consistent with Cs⁺/H⁺ antiport activity of Gdx.

### 3.6 Addition of guanidinium enhances cesium tolerance in E. coli

**Fig. 5:**
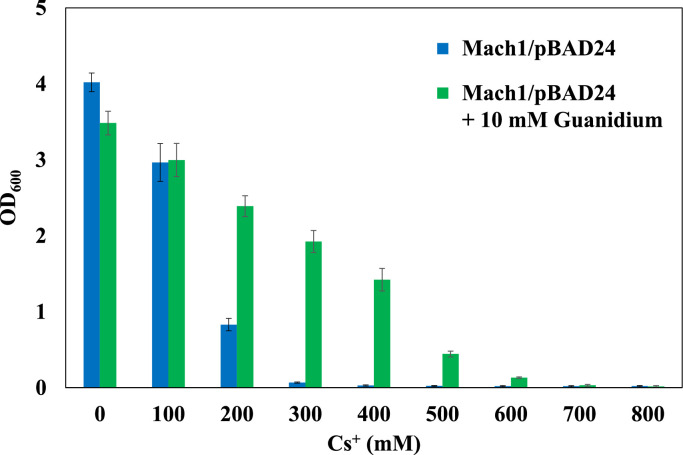
Effect of guanidinium addition on cesium resistance in *E. coli* Mach1/pBAD24. Bar graphs show OD₆₀₀ values of cultures grown for 16 h in LB medium supplemented with various concentrations of CsCl (0–800 mM), with or without 10 mM guanidinium hydrochloride. Blue bars represent conditions without guanidinium (– Gdm⁺), and green bars represent conditions with guanidinium (+ Gdm⁺). Error bars indicate standard deviation from three independent experiments.

To assess the effect of Gdm^+^ on Cs^+^ tolerance, 10 mM GdmCl was added to LB media containing 0–800 mM CsCl, and *E. coli* Mach1™/pBAD24 was cultured under these conditions. In the absence of Gdm^+^, growth was inhibited at 300 mM CsCl. In contrast, under Gdm^+^-supplemented conditions, growth was observed even at 600 mM CsCl, with an OD₆₀₀ of 0.13 after 16 h of incubation. However, at 700 mM CsCl, cell growth was inhibited regardless of the presence of Gdm^+^ (Fig. 5).

### 3.7 Gdx is widely distributed across diverse microbial species

**Fig. 6:**
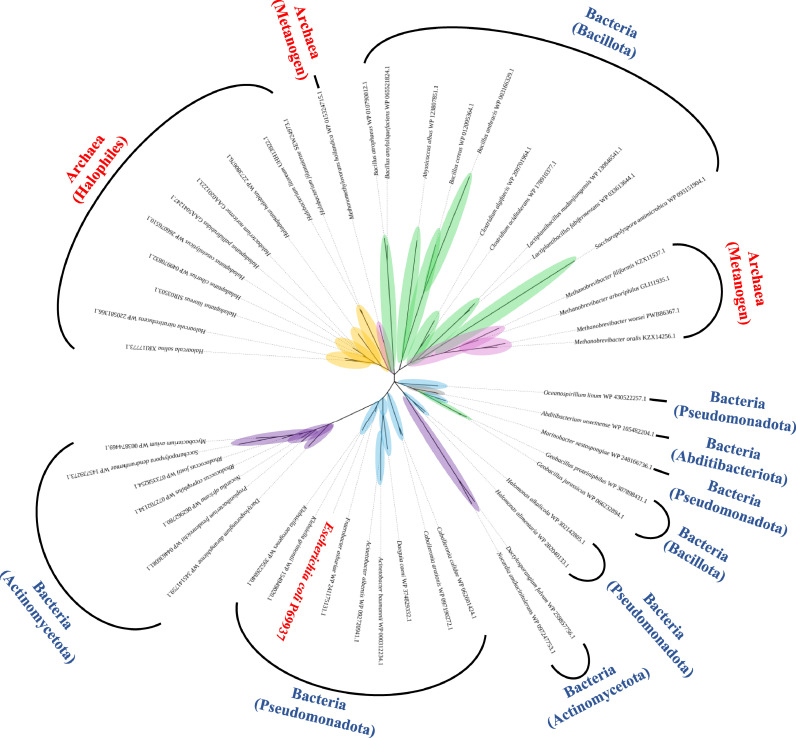
Phylogenetic analysis of Gdx. A phylogenetic tree was constructed based on multiple sequence alignment of Gdx and its homologous proteins. Details are provided in the *Materials and Methods* section. The Gdx from *E. coli* strain K-12 is highlighted in red. Numbers following organism names denote NCBI accession IDs, and the terminal suffix of each label indicates the domain (Bacteria or Archaea).

The multiple sequence alignment and phylogenetic analysis of *E. coli*-derived Gdx and its homologs indicate a broad distribution across bacterial and archaeal taxa (Fig. 6). Lineage-associated clusters are apparent within Proteobacteria, Firmicutes, and several archaeal groups. Homologs were identified not only in *E. coli* and related Enterobacteriaceae but also among actinomycetes, Bacillota (formerly Firmicutes), haloarchaea, and methanogens, indicating conservation of Gdx/SugE-type exporters across diverse prokaryotes.

## 4 Discussion

### 4.1 Transcriptomic characteristics of ZX-1 distinct from its parental strain mach1™

As noted in the Introduction, 11 SNPs and 2 deletions have been identified in strain ZX-1. These mutations likely contribute to the strain-specific phenotypes observed in ZX-1. To generate hypotheses, we performed an exploratory RNA-seq comparison at OD₆₀₀ = 0.4 and describe here effect-size observations (log₂ fold changes; log₂FC) rather than statistical claims (*n* = 1 per condition).

Within this exploratory framework, ZX-1 and Mach1™ displayed distinct expression profiles. Although our subsequent functional analyses focused on the guanidinium exporter *gdx*, other large-effect changes were also noteworthy. For example, *gltS*, encoding a Na⁺/glutamate symporter, showed a large positive log₂FC in ZX-1. Glutamate can act as a compatible solute and is known to utilize K⁺ as a counterion [40,41]. thus, increased glutamate uptake may help stabilize intracellular K⁺ and modestly contribute to Cs⁺ tolerance in *E. coli*.

Genes involved in cysteine metabolism—including *cysI, cysD*, and *cysJ*—also showed large positive log₂FC values. Cysteine and glutamate form γ-glutamylcysteine, a precursor of glutathione, which protects cells from oxidative stress [42]. Notably, γ-glutamylcysteine and glutathione have been reported to enhance Cs⁺ tolerance in *Arabidopsis thaliana* [43]. consistent with a potential protective role for sulfur- and glutamate-linked metabolism.

By contrast, arabinose-pathway genes appeared with large negative log₂FC values in ZX-1. Because the parental and mutant strains carry different plasmid backgrounds (pBAD24 vs pBR322ΔAp), we cannot exclude vector-context effects in this pathway and therefore do not offer mechanistic interpretation for the arabinose metabolism observations in this study. A mutation in *rimK* may still be relevant at the systems level; in *Pseudomonas fluorescens, rimK* deletion has been associated with large-scale proteome remodeling [44].

Under 700 mM CsCl (versus CsCl-free ZX-1; *n* = 1 each), we observed large effect-size increases for the high-affinity K⁺ uptake operon *kdpFABC*. This operon is typically induced when external K⁺ falls below ∼2 mM [45]. and has been reported to be more strongly induced by Cs⁺ than by high Na⁺ [46], consistent with Cs⁺-driven K⁺ depletion as a trigger. We also observed a large positive log₂FC for the ribosome modulation factor *rmf* under Cs⁺ stress. *rmf* promotes 70S dimerization into translationally inactive particles, thereby down-tuning translation and growth [47,48]. Although *hpf* often stabilizes 100S ribosomes under stress, we did not observe a corresponding large effect on *hpf* expression here; *rmf*-only responses under metal-ion stress have been noted previously [49], but stress-specific regulatory details remain unresolved.

Additional large negative log₂FC values were observed for *treB* (the PTS trehalose-specific EIIBC component), which is involved in trehalose phosphorylation and catabolism. Its decrease may reflect a broader osmotic-stress response under Cs⁺ exposure. Likewise, flagellar structural genes showed large negative log₂FC values, aligning with prior observations under high-NaCl osmotic stress [50].

Collectively, these effect-size patterns—together with our functional evidence centered on *gdx*—outline a provisional model in which ZX-1 mitigates Cs⁺ toxicity by supporting K⁺ homeostasis and adjusting translational activity, while sulfur/glutamate-linked metabolism may further buffer cellular stress. These exploratory observations motivate targeted follow-up experiments to define causality.

Based on these results, the following section focuses on the functional aspects of *gdx*, which was suggested to contribute to Cs⁺ resistance.

### 4.2 The guanidinium exporter gdx mediates cs⁺ efflux and contributes to cesium resistance

RNA-seq analysis revealed that the guanidinium exporter *gdx* is specifically upregulated at the transcriptional level in strain ZX-1. *gdx* was selected for investigation because it is known to be involved in the efflux of the monovalent cation guanidinium (Gdm⁺) [39]. The amino acid sequence of *E. coli* K-12 Gdx used in this study was predicted by AlphaFold3 to adopt the characteristic four-transmembrane-helix architecture of the SMR family. Consistently, the membrane topology prediction also indicated four transmembrane segments (Figure S2). These structural features are in agreement with previously reported Gdx structural models [23,24,51]. When *gdx* was cloned into the expression vector pBAD24 (pBAD_Gdx) and introduced into the Cs⁺-sensitive *E. coli* strain Mach1™, the transformed strain exhibited higher Cs⁺ resistance than the Cs⁺-resistant strain ZX-1 upon induction with 2 % (w/v) l-arabinose (Fig. 2). This observation suggests that *gdx* is likely a major contributor to the Cs⁺-resistance phenotype in ZX-1.

To further examine substrate specificity, *gdx* was expressed in *E. coli* KNabc, which lacks the major Na⁺/H⁺ antiporters. Using everted membrane vesicles from this background, Na⁺-coupled antiport activity was not detected (Figure S5). In contrast, weak activity toward K⁺ was observed in both control and Gdx-expressing vesicles, with slightly higher activity in the latter, consistent with very low apparent affinity of Gdx for K⁺.

Notably, Gdx transported Cs⁺, albeit with much lower affinity than for Gdm⁺ (Fig. 3, Table 2). Across pH 8.0–9.0, apparent substrate affinity increased toward pH 9.0 for both ions (Figs. 3, S6, S7). At pH 9.0, the apparent *K*ₘ for Cs⁺ was ∼15.1 mM, whereas that for Gdm⁺ was 29.2 µM, indicating an ∼517-fold lower apparent affinity for Cs⁺ relative to Gdm⁺. These results indicate that Gdx, functioning as an H⁺-coupled antiporter, can recognize and transport Cs⁺ as a secondary substrate despite its low substrate affinity. The ability of Gdx to transport chemically distinct Cs⁺ likely arises from the relaxed substrate specificity and structural flexibility characteristic of SMR transporters. In the following section, we discuss the molecular mechanism underlying this low-affinity Cs⁺ transport.

#### 4.2.1 Mechanistic rationale (hypothesis)

The observation that Gdx exports Cs⁺ despite its distinct chemistry relative to Gdm⁺ can be rationalized by features shared across SMR transporters. Gdm⁺ bears a full positive charge but is only weakly hydrated, lowering the desolvation penalty for entry into a membrane-embedded binding site [52]. Cs⁺ is likewise among the weakest hydrated alkali cations, which facilitates its partitioning into aromatic and hydrophobic cavities [53]. SMR binding pockets—including that of Gdx by homology—are enriched in aromatic side chains, where cation–π interactions and relatively loose electrostatic recognition dominate over strict shape complementarity [20]. We therefore interpret Cs⁺ transport by Gdx as low-affinity adventitious substrate translocation that arises from relaxed substrate recognition and the plasticity of the proton-coupled transport cycle, rather than as a high-efficiency primary specificity. This interpretation is consistent with the ∼517-fold higher apparent affinity for Gdm⁺ than for Cs⁺ observed in this study.

Consistent with this model, intracellular measurements showed that in the control strain Mach1™/pBAD24, intracellular Cs⁺ increased markedly—and intracellular K⁺ decreased sharply—as the external CsCl concentration was raised from 200 mM to 700 mM (Fig. 4). By contrast, ZX-1/pBR322ΔAp and Mach1™/pBAD_Gdx maintained lower intracellular Cs⁺ levels together with higher intracellular K⁺ levels under the same conditions, strongly suggesting that Gdx mediates Cs⁺ efflux and supports maintenance of intracellular K⁺ homeostasis.

To further contextualize these observations, we compared the range of substrates recognized by Gdx with other known Cs⁺ transporters or Cs⁺-binding proteins. Gdx (also referred to as SugE [20,21]) exports the Y-shaped, planar organic cation Gdm^+^ [54]. as well as several quaternary ammonium compounds and confers resistance to tributyltin [22,39,55]. In contrast, the Cs⁺/H⁺ antiporter CshA from *Microbacterium* sp. TS-1, which is absent from *E. coli*, is an MFS transporter with 14 transmembrane helices[12], whereas Gdx is a 105-amino-acid SMR transporter predicted to form a homodimer with four transmembrane helices per monomer [20]. A conserved glutamate at position 13 is essential for Gdm⁺ export by Gdx [20], but how Cs⁺—which lacks structural similarity to Gdm⁺—is recognized and translocated remains unresolved. Notably, halophilic β-lactamases have been reported to selectively bind Cs⁺ via interactions involving a tryptophan side chain and backbone oxygen atoms of glutamine and threonine residues [56]. Expanding the catalog of bona fide Cs⁺ transporters and comparing their sequences and structures may eventually reveal shared motifs or architectural features that underlie Cs⁺ recognition and transport.

In summary, our biochemical and physiological data support a model in which Gdx, a guanidinium/H⁺ antiporter under riboswitch control, can also function as a low-affinity Cs⁺/H⁺ antiporter. In ZX-1 and in Gdx-overexpressing cells, this activity contributes to the maintenance of intracellular K⁺ homeostasis under Cs⁺ stress by limiting Cs⁺ accumulation. However, the detailed structural basis for Cs⁺ binding and translocation by Gdx remains to be clarified in future work.

### 4.3 Induction of the guanidinium riboswitch confers cesium resistance in E. coli

**Fig. 7:**
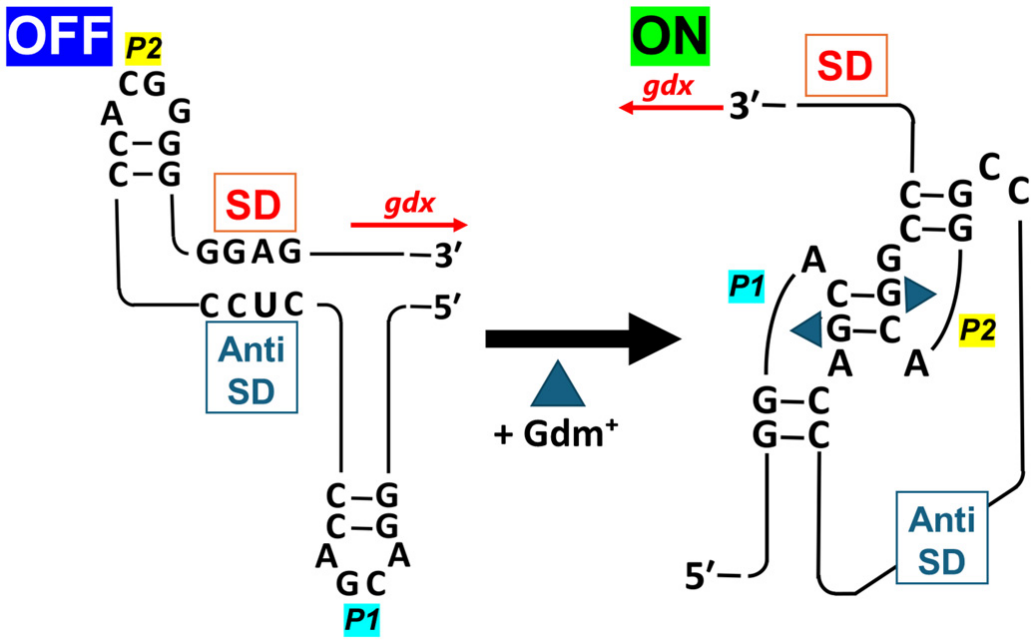
Schematic representation of the guanidinium riboswitch upstream of *gdx*. In the absence of environmental guanidinium, the RNA upstream of *gdx* forms two stem-loops (P1 and P2 loops), which occlude the Shine-Dalgarno (SD) sequence and prevent ribosome binding. In contrast, when guanidinium is present, it binds to guanine residues within the P1 and P2 loops, inducing conformational changes in the secondary structure. This structural rearrangement exposes the SD sequence, thereby enabling translation initiation [57].

The guanidinium exporter Gdx is classified as a member of the small multidrug resistance (SMR) family, but it is known not to be involved in the efflux of antibiotics [20,[23], [24], [25]]. Instead, Gdx has been reported to contribute to the efflux of antimicrobial agents such as guanidinium hydrochloride, benzalkonium chloride, and sodium dodecyl sulfate [19]. Gdx expression is regulated at the translational level by a Gdm^+^-responsive riboswitch located upstream of its ORF. Upon binding of Gdm⁺ to specific guanine bases within the P1 and P2 loops, the RNA secondary structure changes to expose the Shine–Dalgarno sequence, thereby promoting translation (Fig. 7) [20,21,57].

A re-examination of the genomic sequence upstream of the *gdx* ORF in the strain ZX-1 revealed a 20 bp deletion that encompasses the region containing the P2 loop of the guanidinium-responsive riboswitch (Fig. 1). This structural alteration is presumed to result in the constitutive exposure of the Shine–Dalgarno sequence, leading to constant expression of Gdx in ZX-1. The absence of previously reported Cs^+^-resistant ***E. coli*** strains may be explained by the tight translational regulation of *gdx* via its upstream riboswitch in wild-type backgrounds. In contrast, the deletion in ZX-1 disrupts this regulatory mechanism, allowing continuous Gdx production and thereby contributing to its enhanced Cs⁺ resistance.

This study demonstrates that supplementation of the growth medium with Gdm⁺ induces gdx expression via its riboswitch, thereby enhancing Cs⁺ efflux and markedly improving Cs⁺ resistance in *E. coli* (Fig. 5). These findings indicate that gdx, under riboswitch control, functions as an inducible Cs⁺ export pathway.

Homologs of Gdx were identified not only in Gram-negative bacteria but also in Gram-positive bacteria and Archaea (Fig. 6), with lineage-associated clusters apparent across these groups. This broad distribution suggests that uncharacterized Cs⁺-resistance mechanisms may exist in diverse microorganisms.

The results further suggest that microorganisms harboring *gdx* or its homologs could develop inducible Cs⁺ resistance in response to environmental Gdm⁺. This finding raises the possibility that Cs⁺ efflux functions, normally masked by riboswitch regulation, may become active under certain environmental conditions. Notably, a Cs⁺ efflux function that is ordinarily repressed by riboswitch control became evident in strain ZX-1 owing to mutations within the riboswitch region, suggesting that additional genes with latent, riboswitch-silenced functions remain to be discovered.

## 5 Conclusion

In this study, we investigated the Cs⁺ resistance mechanism of *E. coli* strain ZX-1 using RNA-seq analysis. Our results revealed that the guanidinium exporter Gdx, previously known for its role in Gdm⁺ efflux, also unexpectedly contributes to the export of Cs⁺. This finding not only uncovers a previously unrecognized Cs⁺ efflux mechanism in *E. coli* but also highlights a novel functional aspect of Gdx. A limitation of this study is that the precise structural basis for Cs⁺ recognition by Gdx remains unresolved. Further structural and biochemical analyses will be required to identify the Cs⁺-binding determinants within Gdx and to clarify how it recognizes and exports Cs⁺, a substrate with a structure markedly different from guanidinium. In addition, discovery and functional analysis of additional Cs⁺ transporters will deepen our understanding of substrate recognition in transporters.

## Data availability statement

All discussed data are included in the manuscript or in the Supplementary Information. Please contact the corresponding author regarding other requests.

## CRediT authorship contribution statement

**Daiki Kojima:** Writing – review & editing, Writing – original draft, Resources, Methodology, Investigation, Formal analysis, Data curation. **Masahiro Ito:** Writing – review & editing, Supervision, Resources, Project administration, Funding acquisition, Data curation, Conceptualization.

## Declaration of competing interest

The authors declare the following financial interests/personal relationships which may be considered as potential competing interests: Given his role as editorial board member, Dr. Masahiro Ito, had no involvement in the peer-review of this article and has no access to information regarding its peer-review. Full responsibility for the editorial process for this article was delegated to Dr. Qunxin She.

## Appendix Supplementary materials

Image, application 1

Image, application 2

Image, application 3
