# Supplementary material for: Gdx mediates low-affinity Cs⁺/H⁺ antiport and confers cesium resistance in Escherichia coli
Source: Eng Microbiol. 2025 Dec 18;6(1):100251. doi: 10.1016/j.engmic.2025.100251 (PMC13064478; doi:10.1016/j.engmic.2025.100251)
Supplement: Supplementary file 2 [file mmc2.pptx]

## Slide 1
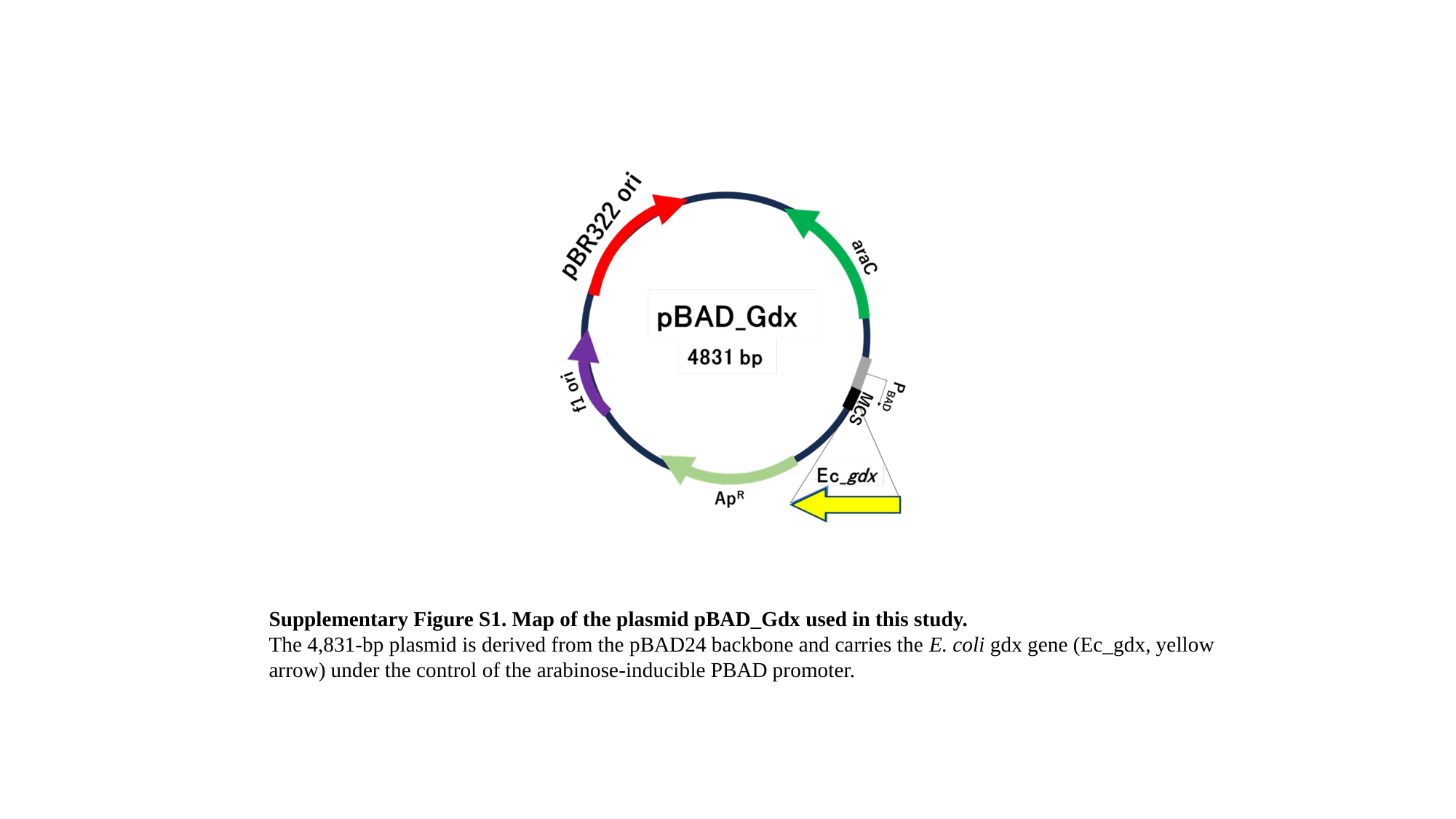

Supplementary Figure S1. Map of the plasmid pBAD_Gdx used in this study.
The 4,831‑bp plasmid is derived from the pBAD24 backbone and carries the E. coli gdx gene (Ec_gdx, yellow arrow) under the control of the arabinose‑inducible PBAD promoter.

## Slide 2
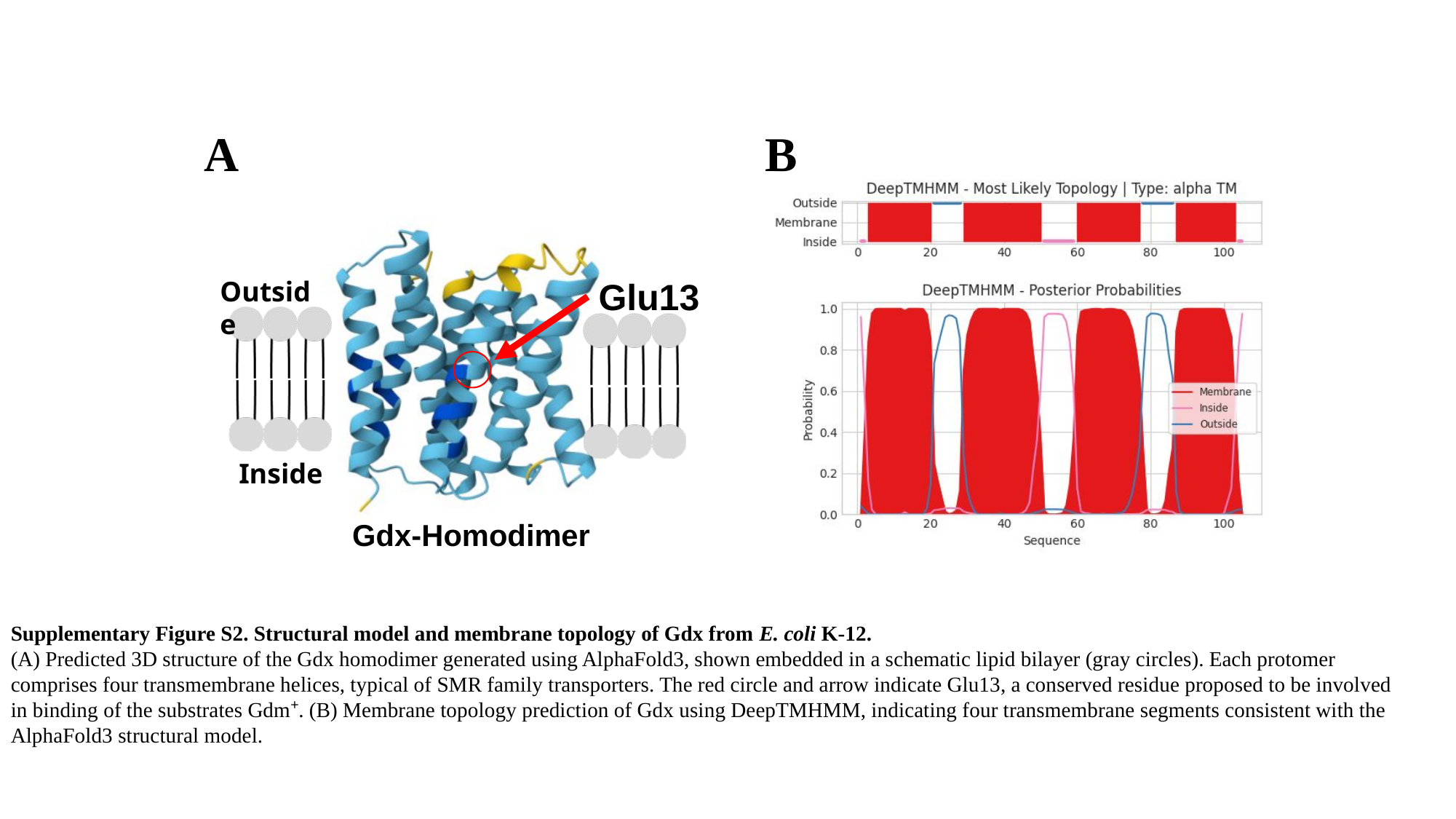

A
B
Glu13
Gdx-Homodimer
Outside
Inside
Supplementary Figure S2. Structural model and membrane topology of Gdx from E. coli K‑12.
(A) Predicted 3D structure of the Gdx homodimer generated using AlphaFold3, shown embedded in a schematic lipid bilayer (gray circles). Each protomer comprises four transmembrane helices, typical of SMR family transporters. The red circle and arrow indicate Glu13, a conserved residue proposed to be involved in binding of the substrates Gdm⁺. (B) Membrane topology prediction of Gdx using DeepTMHMM, indicating four transmembrane segments consistent with the AlphaFold3 structural model.

## Slide 3
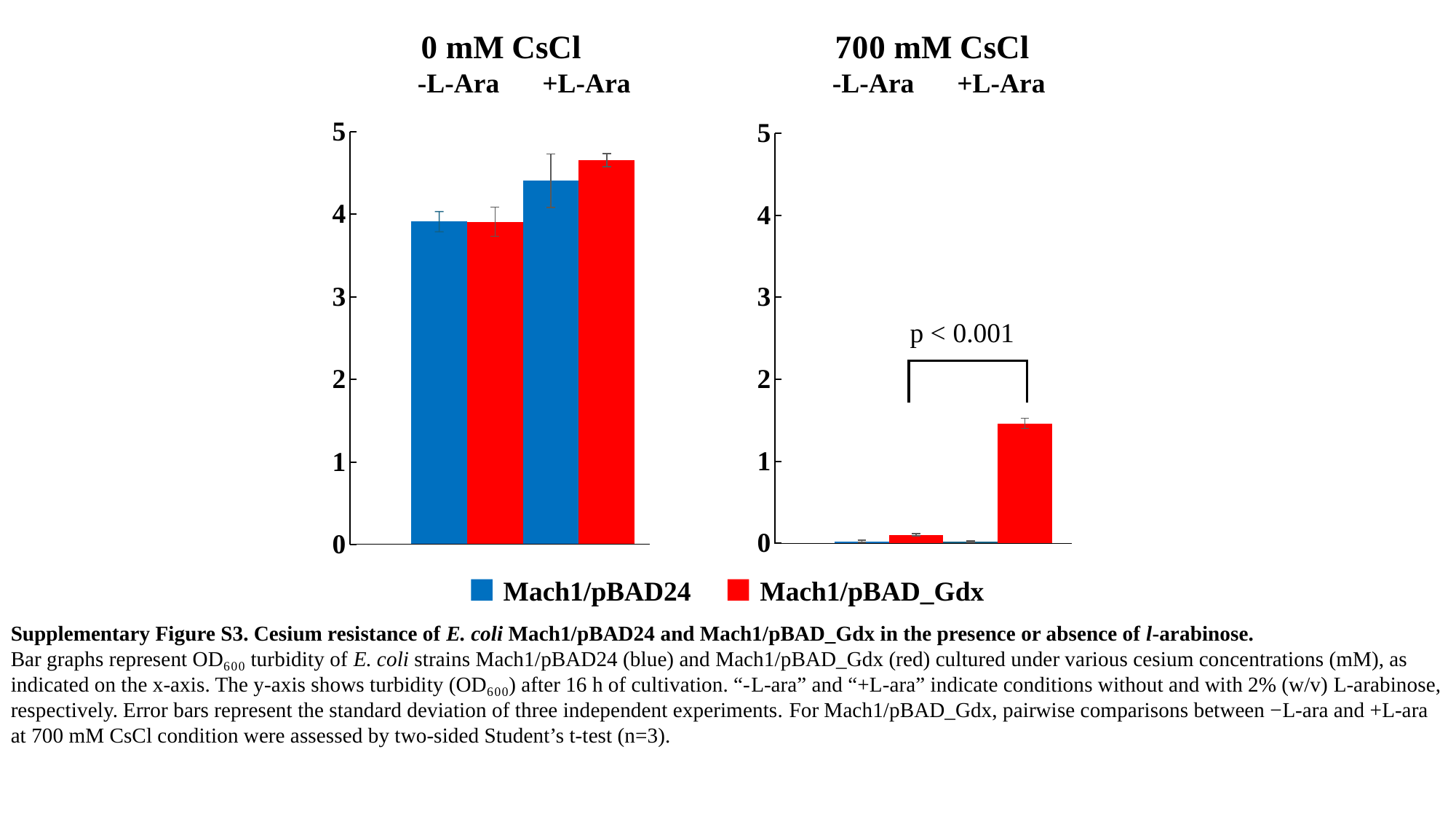

### Chart: 700 mM CsCl
| Category | Mach1/pBAD24 | Mach1/pBAD-Gdx | Mach1/pBAD24(l-Ara) | Mach1/pBAD-Gdx(l-Ara) |
|---|---|---|---|---|
### Chart: 0 mM CsCl
| Category | Mach1/pBAD24 | Mach1/pBAD-Gdx | Mach1/pBAD24(l-Ara) | Mach1/pBAD-Gdx(l-Ara) |
|---|---|---|---|---|
| 0 mM Cs⁺ | 3.91 | 3.908333333333333 | 4.405 | 4.653333333333333 |-l-Ara
+l-Ara
-l-Ara
+l-Ara
p < 0.001
Mach1/pBAD24
Mach1/pBAD_Gdx
Supplementary Figure S3. Cesium resistance of E. coli Mach1/pBAD24 and Mach1/pBAD_Gdx in the presence or absence of l-arabinose.
Bar graphs represent OD₆₀₀ turbidity of E. coli strains Mach1/pBAD24 (blue) and Mach1/pBAD_Gdx (red) cultured under various cesium concentrations (mM), as indicated on the x-axis. The y-axis shows turbidity (OD₆₀₀) after 16 h of cultivation. “-l-ara” and “+l-ara” indicate conditions without and with 2% (w/v) l-arabinose, respectively. Error bars represent the standard deviation of three independent experiments. For Mach1/pBAD_Gdx, pairwise comparisons between −l-ara and +l-ara at 700 mM CsCl condition were assessed by two-sided Student’s t-test (n=3).

## Slide 4
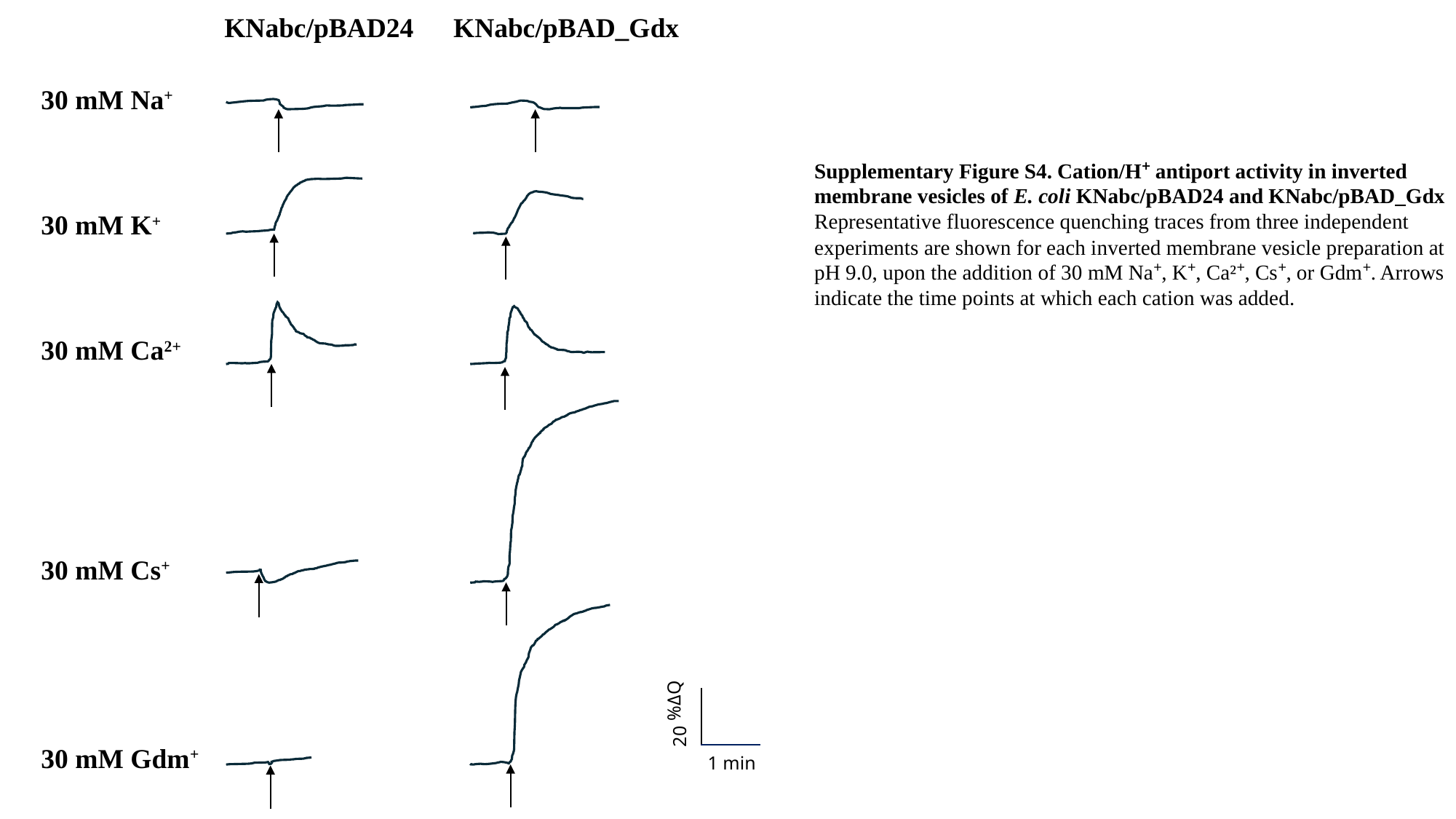

KNabc/pBAD24
KNabc/pBAD_Gdx
30 mM Na+
Supplementary Figure S4. Cation/H⁺ antiport activity in inverted membrane vesicles of E. coli KNabc/pBAD24 and KNabc/pBAD_Gdx
Representative fluorescence quenching traces from three independent experiments are shown for each inverted membrane vesicle preparation at pH 9.0, upon the addition of 30 mM Na⁺, K⁺, Ca²⁺, Cs⁺, or Gdm⁺. Arrows indicate the time points at which each cation was added.
30 mM K+
30 mM Ca2+
30 mM Cs+
%ΔQ
20
1 min
30 mM Gdm+

## Slide 5
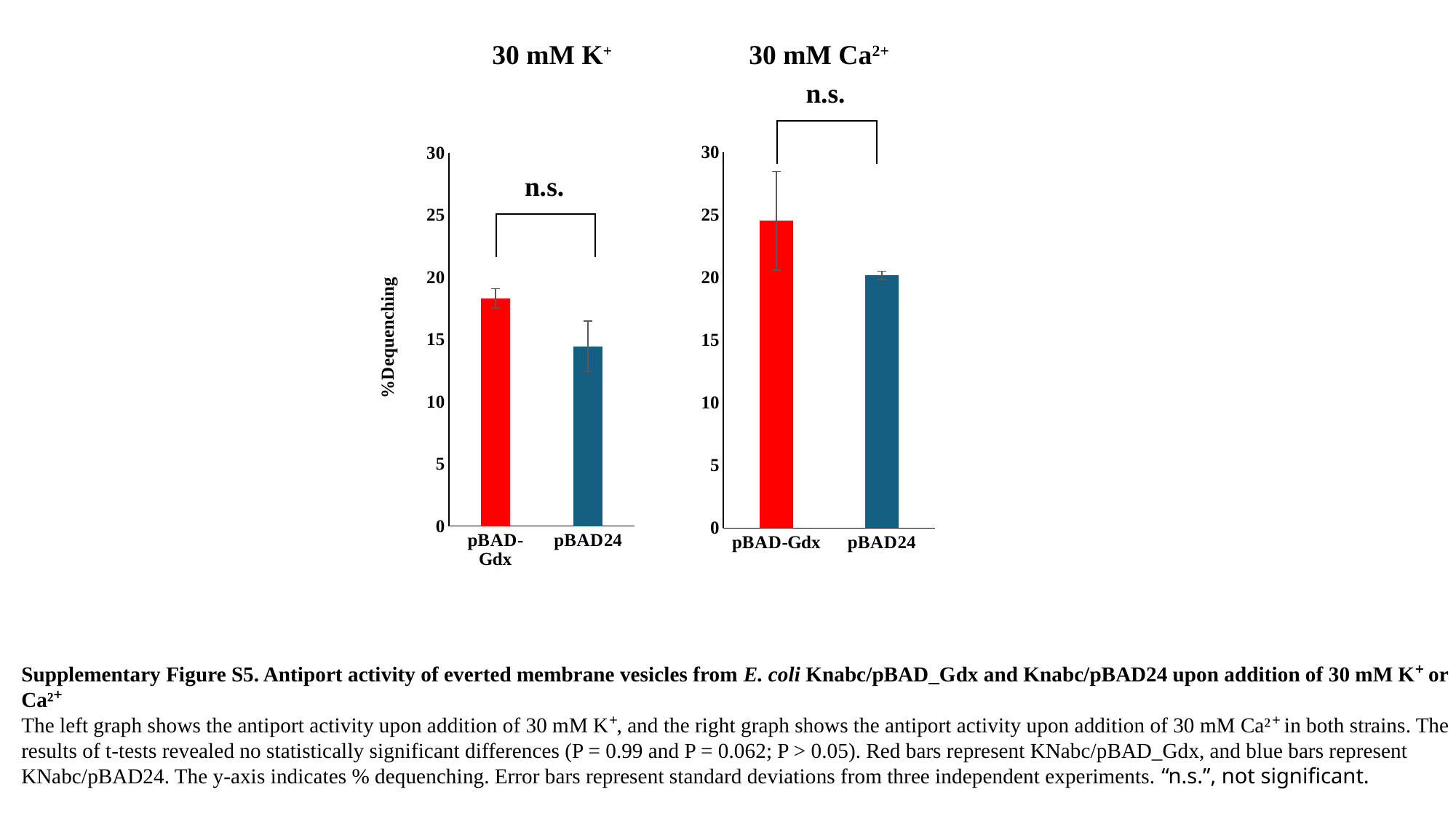

30 mM Ca2+
30 mM K+
n.s.
### Chart
| Category | |
|---|---|
| pBAD-Gdx | 18.283333333333335 |
| pBAD24 | 14.43 |
### Chart
| Category | |
|---|---|
| pBAD-Gdx | 24.526666666666667 |
| pBAD24 | 20.16 |n.s.
Supplementary Figure S5. Antiport activity of everted membrane vesicles from E. coli Knabc/pBAD_Gdx and Knabc/pBAD24 upon addition of 30 mM K⁺ or Ca²⁺
The left graph shows the antiport activity upon addition of 30 mM K⁺, and the right graph shows the antiport activity upon addition of 30 mM Ca²⁺ in both strains. The results of t-tests revealed no statistically significant differences (P = 0.99 and P = 0.062; P > 0.05). Red bars represent KNabc/pBAD_Gdx, and blue bars represent KNabc/pBAD24. The y-axis indicates % dequenching. Error bars represent standard deviations from three independent experiments. “n.s.”, not significant.

## Slide 6
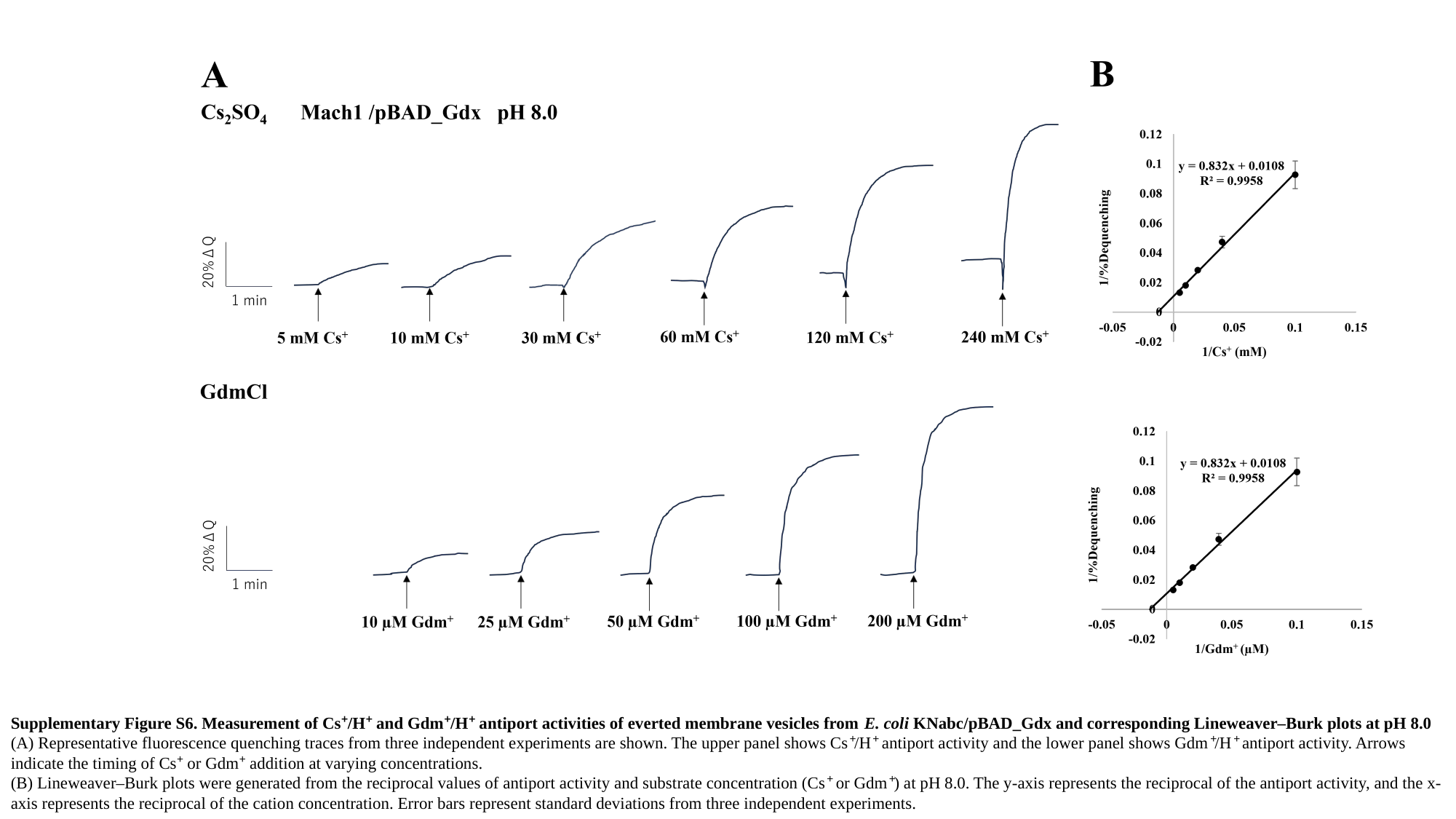

Supplementary Figure S6. Measurement of Cs⁺/H⁺ and Gdm⁺/H⁺ antiport activities of everted membrane vesicles from E. coli KNabc/pBAD_Gdx and corresponding Lineweaver–Burk plots at pH 8.0
(A) Representative fluorescence quenching traces from three independent experiments are shown. The upper panel shows Cs⁺/H⁺ antiport activity and the lower panel shows Gdm⁺/H⁺ antiport activity. Arrows indicate the timing of Cs⁺ or Gdm⁺ addition at varying concentrations.
(B) Lineweaver–Burk plots were generated from the reciprocal values of antiport activity and substrate concentration (Cs⁺ or Gdm⁺) at pH 8.0. The y-axis represents the reciprocal of the antiport activity, and the x-axis represents the reciprocal of the cation concentration. Error bars represent standard deviations from three independent experiments.

## Slide 7
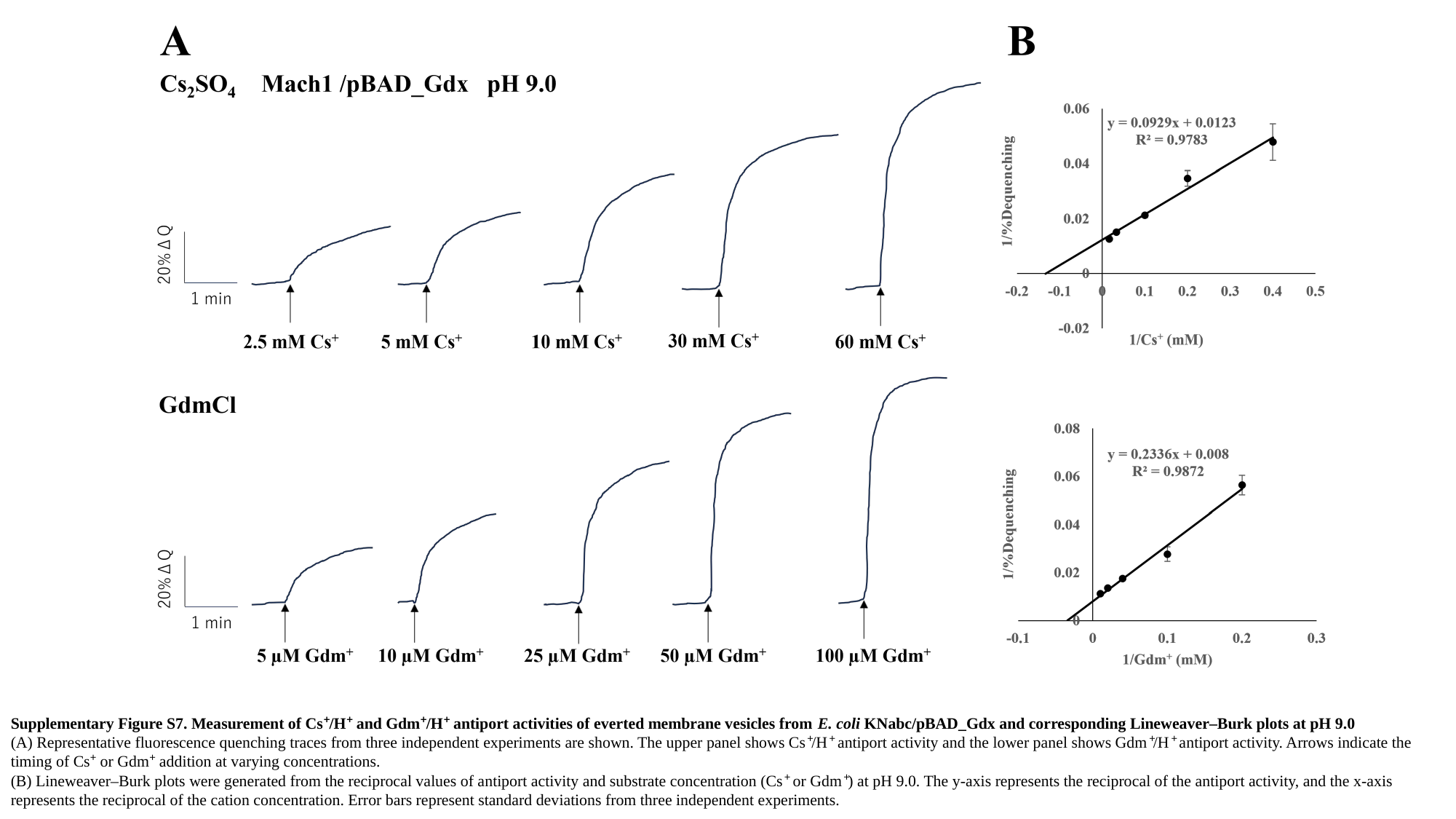

Supplementary Figure S7. Measurement of Cs⁺/H⁺ and Gdm⁺/H⁺ antiport activities of everted membrane vesicles from E. coli KNabc/pBAD_Gdx and corresponding Lineweaver–Burk plots at pH 9.0
(A) Representative fluorescence quenching traces from three independent experiments are shown. The upper panel shows Cs⁺/H⁺ antiport activity and the lower panel shows Gdm⁺/H⁺ antiport activity. Arrows indicate the timing of Cs⁺ or Gdm⁺ addition at varying concentrations.
(B) Lineweaver–Burk plots were generated from the reciprocal values of antiport activity and substrate concentration (Cs⁺ or Gdm⁺) at pH 9.0. The y-axis represents the reciprocal of the antiport activity, and the x-axis represents the reciprocal of the cation concentration. Error bars represent standard deviations from three independent experiments.
