# Supplementary material for: Gdx mediates low-affinity Cs⁺/H⁺ antiport and confers cesium resistance in Escherichia coli
Source: Eng Microbiol. 2025 Dec 18;6(1):100251. doi: 10.1016/j.engmic.2025.100251 (PMC13064478; doi:10.1016/j.engmic.2025.100251)
Supplement: Supplementary file 3 [file mmc3.docx]

Supplemental Table2. Nucleotide sequences of Gdx and its upstream 200 bp in strains Mach1 and ZX-1

>Mach1 (position: 1,155,386–1,155,903, + strand) accession DRA017250

GTGCAGCAACGAAAGCGCAGCAATAAGCAATAACGGTACGACAGTTATGTCGTGCCGTTTGTTTTTTTGCGATAGTCACAAAGGTAATAGTTGAAATTCCCCTGCCACCTGGCAAAATATCCGTTCAACCATCAGCTTTGCAGGACGACCTGCAAACGCCTCTTTTCACCGGGGACGGCCCCAATTCTCCGGAGCCTGAT**ATG**TCCTGGATTATCTTAGTTATTGCTGGTCTGCTGGAAGTGGTATGGGCCGTTGGCCTGAAATATACCCACGGCTTTAGCCGTTTGACGCCGAGTGTTATTACCGTGACGGCGATGATAGTCAGTATGGCGCTACTTGCCTGGGCGATGAAATCGTTACCAGTAGGGACGGCTTATGCCGTGTGGACGGGTATTGGCGCAGTCGGCGCGGCTATCACCGGCATTGTGCTGCTCGGTGAGTCCGCTAACCCGATGCGCCTGGCGAGTCTGGCGTTAATCGTATTGGGGATTATTGGTCTGAAACTCAGCACTCACTAA

>ZX-1 (position: 1,155,386–1,155,903, + strand) accession DRA017249

GTGCAGCAACGAAAGCGCAGCAATAAGCAATAACGGTACGACAGTTATGTCGTGCCGTTTGTTTTTTTGCGATAGTCACAAAGGTAATAGTTGAAATTCCCCTGCCACCTGGCAAAATATCCGTTCAACCATCAGCTTTGCAGGACGACCTGCAAACGCCTCTTTTCACCGGAGCCTGAT**ATG**TCCTGGATTATCTTAGTTATTGCTGGTCTGCTGGAAGTGGTATGGGCCGTTGGCCTGAAATATACCCACGGCTTTAGCCGTTTGACGCCGAGTGTTATTACCGTGACGGCGATGATAGTCAGTATGGCGCTACTTGCCTGGGCGATGAAATCGTTACCAGTAGGGACGGCTTATGCCGTGTGGACGGGTATTGGCGCAGTCGGCGCGGCTATCACCGGCATTGTGCTGCTCGGTGAGTCCGCTAACCCGATGCGCCTGGCGAGTCTGGCGTTAATCGTATTGGGGATTATTGGTCTGAAACTCAGCACTCACTAACTACCGGGCTGCTGTACCCA
